# Taphonomic Trajectory of Diagenesis: How Site Formation Should Inform Biological Sampling Strategies for Isotopic Studies of Ancestors

**DOI:** 10.1002/ajpa.70070

**Published:** 2025-07-02

**Authors:** Melanie M. Beasley

**Affiliations:** ^1^ Department of Anthropology Purdue University West Lafayette Indiana USA

**Keywords:** diagenesis, geoarchaeology, stable isotopes, taphonomy

## Abstract

**Objectives:**

Stable isotope analyses of archaeological bone have become an increasingly common research avenue for interpreting past human behavior. To ensure reliable isotope data is used for interpretations, bone samples must meet quality control standards. The common quality control measures for bone samples rely on post‐sampling destructive approaches. As increasing attention is focused on the ethical use of Ancestors in anthropological research, sampling strategies should prioritize minimal impact to individuals that are less likely to yield viable data for research projects supported by descendant communities.

**Materials and Methods:**

Here, I use previously published stable isotope and diagenesis measures from the Vineyards at Marsh Creek Site (CA‐CCO‐548), supported through tribal consultation and approved by the Most Likely Descendent, as a case study to highlight how geoarchaeology can inform the taphonomic trajectories of individual burials or groups of burials within distinct site formation processes.

**Results:**

Isotope quality control measures indicate that burials located in a context of rapid sedimentation had a higher rate of good bone preservation (100% for collagen and 82% for bioapatite) compared to burials from a stratigraphic context of slow sedimentation in older alluvial deposits (73% for collagen and 63% for bioapatite).

**Discussion:**

Bone preservation corresponds to patterns in site stratigraphy, therefore geoarchaeological data can be used to inform the sampling strategies, rather than relying on post‐processing destructive methods for diagenesis assessment. Sampling strategies should become increasingly reliant on contextual information based on nuanced analyses (i.e., geoarchaeology) of local burial environments to better understand the taphonomic trajectory of bone.

## 1 Introduction

Stable isotope analysis is a method in the archaeological science toolkit used regularly by anthropologists to explore human behavior of past populations (Beasley and Somerville 2023). Advances in instrumentation have moved the capabilities of archaeological scientists from producing a few dozen stable carbon (δ^13^C) and nitrogen (δ^15^N) isotope values from archaeological bone collagen samples across multiple sites (e.g., Walker and Deniro 1986) to researchers regularly producing hundreds of values from single sites to capture population variability (e.g., Bartelink et al. 2020). As stable isotope analysis became routine in archaeological research, a critical aspect of the application of the method was to characterize any diagenetic alteration to ensure that biogenic isotope values were used for interpretations. There has been much research effort toward understanding patterns of diagenesis (Nielsen‐Marsh and Hedges 2000a, 2000b; Nielsen‐Marsh et al. 2007; Smith et al. 2007), improving quality control measures (Beasley et al. 2014; Beasley et al. 2024; Guiry and Szpak 2021; Scaggion et al. 2024), proposing new quality control measures and methods (Chesson, Beasley, et al. 2021; Sponheimer et al. 2019), comparing inter‐laboratory and intra‐individual variation (Berg et al. 2022; Pestle et al. 2014; Chesson, Chau, et al. 2021; Chesson et al. 2019; Edwards et al. 2022), and establishing best practices for reporting isotope data (Roberts et al. 2018; Szpak et al. 2017; Vaiglova et al. 2022). Common criteria for identifying unreliable (diagenetically altered) data, such as percent yields (Ambrose 1990; Chesson, Beasley, et al. 2021), atomic C:N ratios of collagen samples (DeNiro 1985; Guiry and Szpak 2021), and for bioapatite samples measures of crystallinity (IR‐SF) (LeGeros 1981; Weiner and Bar‐Yosef 1990) and carbonate content (C/P) (LeGeros 1981; Wright and Schwarcz 1996), often set standards as a “one size fits all” approach. Guiry and Szpak (2021) argue that for C:N ratios, the quality control criteria should be specific to certain taxa and environments. If quality control criteria are tailored to specific taxa and burial environments, then practitioners should devise sampling strategies that maximize samples of Ancestors with the greatest potential to yield reliable isotope values for interpretations. The aim of this paper is not to rehash existing concerns and approaches to characterizing diagenesis, but rather to explore the importance of sampling strategies informed by the archaeological context of the burial environment, specifically starting with geoarchaeological analysis of the site formation to understand the taphonomic trajectory of bone collagen and bioapatite that results in diagenetic alteration to stable isotope values. Here the classic taphonomic problem of how does the geosphere alter biogenic isotope values when bone ceases to be regulated by physiological mechanisms in the biosphere, starts with the assumption that the site formation processes during and after human occupation of a site will predict the diagenetic quality control measures.

## 2 Background

### 2.1 Destructive/Instructive Analysis of Ancestors

First, a note on the intentionality of the use of two terms, Ancestors and instructive analysis. The use of “Ancestors” to refer to the remains of individuals from archaeological contexts follows suggested best practices in recent collaborative discourse on working with descendant communities in North America (Bader et al. 2023; Bardill et al. 2018; Blakey 2022; Martinez 2006; Meloche et al. 2021). As biological anthropology is in the midst of a paradigm shift in the ethical standards for professional practices related to research, teaching, community engagement, and curation involving deceased individuals from historically documented collections (de la Cova et al. 2024), purchased skeletal anatomy collections from India (Agarwal 2024), and collections of Native American Ancestors in universities and museums (Stantis et al. 2023; Teeter et al. 2021), it is critical to use terminology that combats the commodification and objectification of individuals as scientific objects. Following suggested best practices, Ancestors is used in place of “skeletal material”, “human remains”, or other variants, while the term “individual(s)” and “burial(s)” are still used where appropriate as both terms reference the individuality of a person. Second, the suggestion of the use of *instructive analysis* rather than *destructive analysis* is to recognize a move toward collaborative community‐lead research that puts anthropology to work for tribal communities. The use of *destructive analysis* has negative connotations of extractive and harmful processes, which has been true for much of anthropological research. The use of *instructive analysis* is a recognition of the paradigm shift in the discipline that prioritizes community engagement and partnerships with descendants. As the discipline continues to engage with discourse on how to handle legacy data when modern practices of consultation were not employed, the distinction between *destructive analyses* and *instructive analyses* of biological samples is one possible avenue to recognize work that is rooted in community engagement with tribal‐supported research. The suggestion of the use of the term *instructive analysis* is not intended to be misleading, that the analysis is not still a destructive method, but rather that the choice to use a destructive method was selected by the descendant community to produce knowledge that is instructive and informative to community goals. Both terms are used in the text where appropriate.

The perspective on the treatment of Ancestors and modern deceased individuals is not a human universal and varies by country (de Tienda Palop and Currás 2019, 26). One end of the extreme, is the thought that Ancestors are considered dehumanized biological material as objects of study (Jones and Harris 1998; Rajala 2016), while the other end of the spectrum could be seen as the perspective that efforts should be made to restore individual personhood (de la Cova et al. 2024). These perspectives are so varied that international perspectives in “The Dignity of the Dead: Ethical Reflections on the Archaeology of Human Remains” include statements such as “the study of human remains from Antiquity does not seem to provoke many controversies”, which might hold true for some European countries but is far from the reality experienced by American archaeologists (de Tienda Palop and Currás 2019, 27). While international forensic humanitarianism has engaged in discourse about the rights of deceased individuals for decades (see summaries in Moon 2014, 2019), universities, museums, and professional associations in the United States are now addressing the ethical considerations of the treatment of the thousands of individuals that are curated without consent (Auerbach and Jackson 2022; Brandt et al. 2022; Cornwall et al. 2024; de la Cova 2019, 2020; Stantis et al. 2023; Watkins 2018; Zuckerman et al. 2021). While current federal laws like the Native American Graves Protection and Repatriation Act (NAGPRA) and African American Burial Grounds Preservation Act (AABGPA) provide protections for some deceased individuals, non‐federally recognized tribes are not allowed to seek repatriation. The California Native American Graves Protection and Repatriation Act of 2001 (CalNAGPRA) does allow for repatriation to non‐federally recognized tribes and the 2023 California Assembly Bill 389 implements further restrictions and oversight on the handling, maintenance, and repatriation of Ancestors and cultural items for the purposes of teaching and research.

Over the past few decades, an increasing number of academic and cultural resource management projects have allocated funding to use stable isotope analysis of Ancestors to address research questions concerning paleodiet, paleoenvironment, and migration patterns of prehistoric populations. In California, Indigenous community support for geochemical analysis of Ancestors is variable, with some tribes like the Muwekma Ohlone Tribe of the San Francisco Bay Area permitting *instructive analysis* for collaborative stable isotope and aDNA research (Gardner et al. 2018; Monroe et al. 2022; Panich et al. 2024; Severson et al. 2022). Community‐based research with Indigenous communities puts archaeology to work for tribes and recognizes the connection between living people and the past for the benefit of community goals (Lippert 2006; Martinez 2010; Panich et al. 2024; Teeter et al. 2021). Here, I present a case study to illustrate how sampling strategies and taphonomic trajectories of diagenesis can be informed using geoarchaeological analysis of site formation processes. In consultation with tribal Most Likely Descendant support (i.e., the legal authority designated by the California Native American Heritage Commission with respect to Ancestor remains), the Vineyards at Marsh Creek Site (CA‐CCO‐548) was studied during a construction project mitigation, and all data used in this case study have been previously published for diagenesis research (Beasley et al. 2014; Beasley et al. 2024), stable isotope interpretations (Bartelink et al. 2020), and produced a cultural resource management archaeological report (Wieberg 2010).

### 2.2 Characterization of Bone Diagenesis for Stable Isotope Analysis

A critical step in the method to ensure that measured stable isotope values preserve *biogenic values* is to evaluate possible diagenesis alteration of a sample to demonstrate that the pretreatment protocol has removed any exogenous contaminants. As bone is a biphasic tissue with an organic (collagen) and inorganic (bioapatite) fraction used for stable isotope analysis, both fractions need to be evaluated for diagenesis independently (see Beasley et al. 2024 for discussion of bone diagenesis). Any diagenetic alteration to either phase will be impacted by time, scale, and sequence. The resulting isotope value has many input variables from the burial context that can change the trajectory of the final measured value despite it being a “stable” isotope, so data quality control measures are critical to ensure reliable results.

For bone collagen, assessment of C:N ratios (DeNiro 1985; Guiry and Szpak 2021) and the percent yield of bone collagen after demineralization (Ambrose 1990) are both common quality control methods. Both methods require destructive analysis of the bone sample, with the percent yield > 1% used as a threshold after sample preparation to continue to the analysis and C:N ratios used to ensure measurement values are acceptable if between a range of 2.9 to 3.6 (the exact upper limit has been variably reported, see Guiry and Szpak 2021). While both quality control measures are standard post‐processing diagenesis checks, recent studies have proposed non‐destructive methods to target samples with good bone collagen preservation prior to sampling using near‐infrared spectroscopy (NIR) (Sponheimer et al. 2019) or NIR coupled with hyperspectral imaging (HSI) with a chemometric model (Malegori et al. 2023). Portable NIR and HSI are exciting new methods to provide chemical mapping of collagen content prior to sampling, but these do not replace the later diagenesis quality control measure of C:N since the presence of collagen alone does not ensure biogenic values are not altered.

For bone bioapatite, the percent yield of bone bioapatite after removal of the organic phase (Chesson, Beasley, et al. 2021; Chesson, Chau, et al. 2021), the infrared splitting factor (IR‐SF, a measure of crystallinity) (LeGeros 1981; Weiner and Bar‐Yosef 1990), and the carbonate content (C/P) (LeGeros 1981; Wright and Schwarcz 1996) are all measures to assess diagenesis independent of the pretreatment of bone samples to remove exogenous contaminants. Pretreatment of bone samples to remove diagenetic contaminants has included heating a sample in an oxygen atmosphere, rapid treatment with strong acids, and slower treatment with weak acids (see Beasley et al. 2024 for review of literature). However, pretreatment is not always successful, so IR‐SF and C/P are regularly evaluated from infrared or vibrational spectroscopy, x‐ray diffraction, or Raman hyperspectral imaging. The case study samples in this paper were treated with a weak acid and infrared spectroscopy, specifically Fourier transform infrared spectroscopy (FTIR) was used for sample quality assessment (Bartelink et al. 2020).

To better understand the taphonomic trajectory of diagenesis after death as the body transitions from the biosphere to the geosphere within a site, it is key to understand how a site is formed during and after human occupation to contextualize how the geosphere impacts a body at an elemental scale. As geological processes form sites of human occupation later recovered by archaeologists, the Ancestors buried within the sites act as the intersection of multiple processes of burial, decomposition, and fossilization that determine the preservation of the individual. Bone transitions from living tissue to fossil through the process of replacement of the organic phase in bone with the reorganization and recrystallization of the mineral structure of bone impacted by the surrounding burial environment leading to the preservation of the bony structure in an inorganic phase. During the taphonomic trajectory of diagenesis, the body is central as the locus of experience for alteration from the biosphere to the geosphere. The body and this transition might be impacted by biosocial changes of how the body is treated after death by the living through particular funerary rites and burial practices that affect diagenesis. While a site is actively occupied, cultural practices in the form of burial rituals, traditions, treatment of the body after death, and placement of the burial within a site are all initial decisions made by the living that start a body on a particular diagenesis trajectory that can result in alteration to either the collagen or bioapatite. After human occupation of a site has ceased, continued geological processes of sedimentation will impact the site formation by accumulation of sediment to bury a site at varying rates or at the other end of the extreme leaving a site exposed with no further sedimentation. The biphasic nature of bone means that as both phases transition to the geosphere they intersect with the surrounding environment during the continued site formation process until excavated by archaeologists. While this discussion is particularly focused on the diagenesis assessment of human burials, it is recognized that site formation processes and the impact of human occupation at a site will influence the taphonomic trajectories of all biological materials, such as fauna and botanical remains, and should be considered during sample selection.

Current approaches to pre‐screening bone samples for isotope analysis or post‐treatment/post‐preparation assessment often rely on laboratory methods that are divorced from the archaeological/burial context. In most cases, objective and deductive approaches to understanding diagenesis would seem to make most scientific sense. However, I argue that the accumulation of decades of research about how diagenesis impacts stable isotope values lends itself to an interpretation that subjective and inductive approaches that prioritize the context of the site formation, soil type, and ground water permeation, and make central the different intrinsic properties of bone (i.e., size, shape, porosity, etc.) are as important to consider when evaluating the taphonomic trajectory of a bone sample. I argue that considering how geoarchaeology and bone biology impacts diagenesis is more than thinking about the mortuary/funerary deposition decisions made by living individuals acting out particular funerary rites. By taking a holistic approach within the context of site formation, practitioners of biogeochemical analyses should create sampling strategies rooted in geoarchaeological analysis of a site and specific bone biology of elements to evaluate Ancestors within their burial environment. In essence, the taphonomic trajectory could be described as the death experience of an Ancestor's physical body starting with treatment after death by the living and a continuation through site formation processes prior to excavation by archaeologists. This brings the question of diagenesis back to the context in which the individual was recovered to understand if biogenic values are likely to be obtained from geochemical studies. Stable isotope practitioners must continue to diligently consider how to select samples for *instructive analysis* during the study design phase.

## 3 Case Study: The Vineyards at Marsh Creek Site (CA‐CCO‐548)

The Vineyards at Marsh Creek Site (CA‐CCO‐548) is located in present day Brentwood, California in the eastern part of Contra Costa County in Central California. The west side of Marsh Creek is also known as the Pearl Site, while the east side of the site is identified as the John Marsh House locus (CA‐CCO‐18/H) (Wieberg 2010). The west side of the site was the main locus for burials recovered during the cultural resource management project and is the main interest of this case study example. The archaeological mitigation of the site was the result of a proposed residential development. California State Parks, Holman & Associates, Far Western and UC Davis all contributed to various phases of the mitigation process (Wieberg 2010) and tribal support for stable isotope research was approved by the Most Likely Descendant (Bartelink et al. 2020).

Excavations at CA‐CCO‐548 provide material culture evidence of intense occupation of the site over two millennia during the middle part of the Holocene, with AMS dates on 148 burials ranging between 5420 and 2975 cal BP (Eerkens et al. 2013). The site contains a large quantity of faunal and archaeobotanical remains, ground stone artifacts, fire‐affected rock, house floors, storage and cooking features, and hundreds of human burials (Wieberg 2010). The site can be divided into two general areas, one a likely residential occupation locus and the other a cemetery locus within the site boundaries (Wieberg 2010). These two areas of the site showed a higher concentration of features identified in the west side of the site versus a denser concentration of burials in the east side of the site. The geoarchaeological analysis of the site concluded that Marsh Creek, at some point in the past, ran further to the north of the older alluvial deposit. The mounded deposit acted as a barrier for the rapid accumulation of soil in the eastern side of the site. This means that the western side of the site was subject to residential living and midden accumulation, while the eastern side of the site did not accumulate a thick sediment layer or midden deposit over the older deposits. The stratigraphy and site history support the conclusion that burials from the eastern side would be more likely to be affected by diagenesis. The majority of the poor bone preservation samples were excavated from the eastern side of the site, as well as most of the burials not sampled for isotope analysis. Burials that were not initially sampled for isotope analysis were excluded because the visual inspection of the burial suggested that there would be no viable collagen available for extraction. The fact that most of the burials not sampled or burials sampled but with poor bone preservation came from one locus further supports the conclusion that geoarchaeological analysis of site stratigraphy and sediment formation can provide valuable data when designing a sampling strategy for isotope analysis.

Bone collagen and bioapatite stable carbon and nitrogen isotope data from 248 individuals indicate the dietary importance of terrestrial C_3_‐resources and freshwater fish (Bartelink et al. 2020). Of the 248 Ancestor burials sampled, 198 burials had good bone collagen preservation with collagen yield above 1% and C/N ratios between 2.9 and 3.6 (Bartelink et al. 2020). Carbonate to phosphate ratio (C/P) and infra‐red splitting factor (IR‐SF) were calculated using Fourier transform infrared (FTIR) spectroscopy for 232 bone apatite samples and reported in detail elsewhere (Bartelink et al. 2020; Beasley et al. 2014; Beasley et al. 2024). Of the 232 bone apatite samples, 169 samples had good bone bioapatite preservation with IR‐SF values ≥ 3.5 and 87 samples with C/P values ≤ 0.15 (Bartelink et al. 2020). However, at the site neither C/P nor IR‐SF correlated with bioapatite δ^13^C values, which indicates no systematic change to the δ^13^C values. Two examples from the site will be used to demonstrate how site stratigraphy, site history, and the principles of sediment formation can be used as an indicator of burial diagenesis to in turn impact the sampling strategies for isotope analysis.

### 3.1 Site Formation Impacts on Diagenesis

**FIGURE 1:**
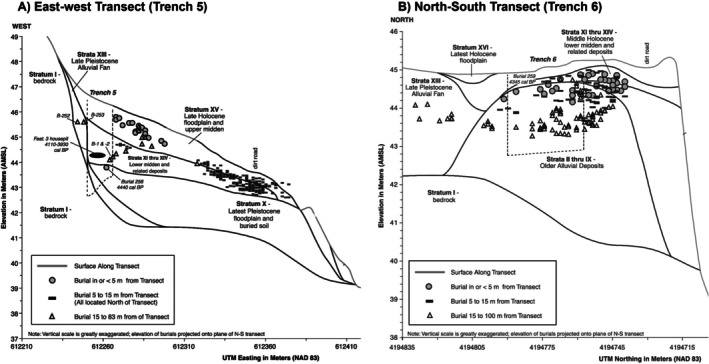
The geoarchaeological analysis of the (A) east–west transect (Trench 5) with discrete packages of sedimentation compared to the (B) north–south transect (Trench 6) with an older alluvial deposit. Each transect shows the relationship of the site sedimentation to the burials in relationship to distance from the transect. (Figured adapted from Meyer 2010 with permission from Far Western Anthropological Research Group.)

By examining the site formation processes of two trenches at Marsh Creek (CA‐CCO‐548) (Meyer 2010), it was determined that the different geologic depositional histories within the site could provide critical information on why the diagenesis trajectories of collagen and bioapatite samples from one site could be drastically different (Figure 1). Meyer (2010) identified an older alluvial deposit in the north–south transect trench that, through erosion, resulted in the formation being mounded in localized areas of the site. The older alluvial deposit displayed traits associated with prolonged weathering and considerable age, including the presence of gypsum or sulfur, suggesting in situ diagenetic alteration of the sediment (Meyer 2010). The significance of this mounded, older alluvial deposit from the middle Pleistocene is that its stratigraphic position suggests that portions of the formation remained on the surface for human use well into the Middle Holocene (Meyer 2010). Any Ancestors buried in the formation were not protected by further sedimentation accumulating on top of the burials, which would provide further potential protection from diagenesis. Therefore, it would be expected that Ancestors with compromised bone would be more likely to be found within the older alluvial deposit.

Meyer (2010) also analyzed the east–west transect which provided a different sedimentation history to compare to the mounded older alluvial deposit. The east–west transect stratigraphy supported a faster accumulation of sediment formation because discrete packages of sedimentation known as fining upward packages were identified (Meyer 2010). Fining upward packages are related to fluvial sequences such as migrating channels or overbank deposits resulting from flood events, which are identified by coarse material at the bottom of the package and finer material at the top (Friedman and Sanders 1978). The stratigraphy of the east–west transect identified multiple fining upward packages suggesting that the process occurred quickly and that sedimentation happened faster than soil formation (Meyer 2010). This means that burials from this area of the site were buried and quickly covered by new packages of sediment, which would have resulted in better protection from diagenesis of the Ancestors.

The most reliable geoarchaeological data derive from these two trenches. To assess the impact of site formation processes on the taphonomic trajectory of each Ancestor, the bone preservation scores (i.e., collagen yield, C:N, IR‐SF, and C/P) of burials within five meters of the trenches are compared to see how well site formation and stratigraphy can predict bone preservation. There were 38 Ancestor burials within five meters of Trench 6. Of those, 30 were sampled for isotope analysis and eight of the samples (27%) did not meet quality control standards for bone collagen, with four samples that had either no remaining bone collagen to be extracted or high C:N values (see Supporting Information for raw values). A minimum of 11 bioapatite samples (37%) had values that did not meet the quality control standards of FTIR measures calculated from either KBr Pellet, ATR, or DRIFT preparations (Beasley et al. 2014). If France et al. (2020) is used as the standard for FTIR‐ATR and FTIR‐DRIFT quality control cutoffs, then 14 (47%) samples have unacceptable IR‐SF values and 22 (73%) samples have unacceptable C/P values. As FTIR is a semi‐quantitative method and quality control values specific to sites and instruments are variable (Guiry and Szpak 2021), the modern bone value range in Beasley et al. (2014) will be used for the interpretation. Of individuals buried near Trench 6, there are 16 (53%) individuals with burial environment taphonomic trajectories that resulted in diagenesis of their bone collagen, bone bioapatite, or both phases of bone. Although not all of the burials excavated from within the older alluvial deposit had compromised bone preservation, the burials from that context were more likely to yield unreliable isotope samples.

There were 27 Ancestor burials within five meters of Trench 5. Of those, 22 were sampled for isotope analysis and all of them had good collagen preservation with four samples (18%; Burials 6, 141, 476, and 479) having possibly compromised bioapatite (see Supporting Information for raw values). As expected, the burials located in the context of rapid sedimentation had a higher rate of good bone preservation (100% for collagen and 82% for bioapatite) resulting in reliable stable isotope values compared to burials from a stratigraphic context of slow sedimentation in the older alluvial deposit (73% for collagen and 63% for bioapatite).

### 3.2 Differential Diagenesis Trajectories of a Specific Stratigraphic Layer

A more specific comparison between the bone preservation of Ancestor burials within five meters of Trench 5 and 6 can be exemplified by comparing the Lower Midden burials. The Lower Midden (4300–3500 cal BP) had three burials that were sampled for isotope analysis. Two Ancestor burials (258 and 259) from Trench 6 and one Ancestor burial (256) from Trench 5 buried in the oldest layer of the site (the Lower Midden), provide an example of how different depositional processes occurring in one site can alter the preservation of isotope bone samples despite being from the same chronological context. The two Ancestor burials in the older mounded alluvial deposit that did not have significant sedimentation accumulation had compromised collagen. Ancestor burial 258 had a low collagen yield, so the sample was not analyzed, while Ancestor burial 259 had a sufficient collagen yield to analyze the sample. After IRMS analysis, Ancestor burial 259 had a compromised C:N value (9.6) and significantly different δ^13^C_col_ (−25.5‰) and δ^15^N (+0.4‰) values from the means of the population, −19.8‰ and + 9.5‰, respectively. Ancestor burial 256 in the rapidly accumulating fining upward packages had excellent preservation of both bone collagen and bioapatite. Compared to Ancestor burial 259, Ancestor burial 256 was equal or within one standard deviation to the population mean values for bone collagen (δ^13^C_col_ = −19.8‰; δ^15^N = +9.8‰) and bioapatite (δ^13^C_apa_ = −14.5‰ vs. population mean δ^13^C_apa_ = −14.0‰). Therefore, the geoarchaeological predictions for each burial context corresponded to the resulting quality control measures.

Although all three burials were chronologically similar, the diagenesis trajectories for each phase of bone were different within areas of the site that had varying depositional processes. Nielsen‐Marsh et al. (2007) found that soil chemistry significantly affects the preservation pathway characterized by the loss of the mineral phase of bone. Time, site depositional processes, and bone biology properties of varied skeletal elements are variables that need further investigation for a better understanding of their relationships with diagenesis trajectories affecting bone samples. Diagenesis is not a process relegated to only fossil samples from the Eocene through the Pleistocene but is a concern for practitioners working in the Late Holocene.

In the Marsh Creek example, the opportunity to sample a large Ancestor population for stable isotope analysis (Bartelink et al. 2020) with tribal community support provided a unique opportunity to investigate factors that influence bone preservation, which has implications for research design when using stable isotope analysis as an instructive methodological tool for archaeological inquiry. If bone preservation corresponds to patterns in site stratigraphy, then geoarchaeological data can be used to inform the sampling strategies prior to any *instructive analysis* of Ancestors. Future study designs supported by tribal community partners should consider not just newer analytical techniques, but what site formation processes can reveal about the likelihood of sampling Ancestors that will yield in vivo isotope values when funding might be limited and meeting ethical standards of our profession to do no harm.

## 4 General Recommendations for Sample Selection

We are often too siloed in our disciplinary specialty and/or regions of study to consider the broad implications across international borders. In my original formulation of this case study, I framed it with a focus on practitioners working within the United States under laws like NAGPRA and CalNAGPRA. While specific laws, customs, and attitudes about research with Ancestor remains vary in international contexts, my aim with presenting the Vineyards at Marsh Creek Project case study is to highlight the need to not solely rely on *post‐processing* diagenetic checks. Non‐destructive methods, such as NIR or NIR‐HIS, are one avenue forward to guide sample selection of Ancestors with the most likely outcome for yielding biogenic isotope values. Here, I recommend that reliance on equipment that might be highly specialized or cost‐prohibitive is not the only solution, but rather we should return to our roots as archaeologists and collaborate with geoarchaeologists assessing site formation processes. Collaboration with geoarchaeologists is not a recommendation limited by geopolitical boundaries, but rather one that all international scholars can apply in their region and at specific sites.

Admittedly a singular case study in California working with a descendant community supportive of *instrucutive* methods where geoarchaeology was funded as part of the original cultural resource management project is not going to be easily operationalized in every context. Geoarchaeological evidence pertaining to site formation processes *should* inform sampling strategies for isotope analyses seems reasonable in theory. But in practice, many practitioners are often in the lab or museum and far removed from the site or other variables uncovered during excavation, mainly the geoarchaeological analyses. In many museum and legacy collection contexts, practitioners select samples based on visual assessment of assumed sample bone quality only to check diagenesis using post‐processing methods. The call to action here is that consumers of stable isotope analysis as a destructive method should prioritize geoarchaeological assessment of site formation processes when performing instructive analyses of all types (i.e., DNA, proteomics, or isotopes) on biological samples (i.e., Ancestor, fauna, or botanical remains). This is a reminder that we have the skills within our discipline of subspecialties in archaeology to collaborate. As best practice when designing new research projects or when working with legacy museum collections it *is possible* to use geoarchaeological data where available to guide sample selection. It might be admittable hard to operationalize in practice at some sites, but when non‐destructive pre‐screening methods are unavailable it should be an option to rely on. Understanding site formation is just one variable in the milieu of variables that can impact the taphonomic trajectory of an individual burial or site (Nielsen‐Marsh and Hedges 2000a, 2000b; Nielsen‐Marsh et al. 2007; Smith et al. 2007). Geoarchaeological data is not just for selection of samples in isotope studies, but also DNA and proteomic research (Kontopoulos et al. 2020). There is not a “one size fits all” solution of how to operationalize sample selection, but the point I emphasize here is *collaboration with geoarchaeologists* to understand site formation processes is one path forward.

## 5 Conclusion

In conclusion, the Vineyards at Marsh Creek Project provided a case study for evaluating the usefulness of geoarchaeological analyses to inform sampling strategies for isotope analysis. In a climate of limited funding for cultural resource management projects and ethical concerns about destructive methods (Stantis et al. 2024), it has become ever more important to be the least destructive when sampling biological tissues and stretch funding resources to maximize the data gained by archaeological research supported by tribal community partners. Stantis et al. (2024), emphasize that ethical applications of isotope analysis should include “working with descendant communities and other rights holders, choosing methods, creating and sharing data, and working mindfully within academia”, but their synthesis of assessing sample quality (i.e., diagenesis) focuses only on post‐sampling and destructive methods. This case study suggests that principles of sedimentation and the resulting stratigraphy interpreted by geoarchaeologists, as well as the site history and residential use patterns of a site, can be valuable gross indicators of bone preservation that aid in the selection of the most likely isotopically viable bone samples before sampling occurs. Evaluation of the taphonomic trajectories of bone diagenesis should not be a “one size fits all” approach as Guiry and Szpak (2021) highlighted when applying C:N ratios as a quality control measure for bone collagen. Study design and sample selection should start with the geoarchaeological context when working with Ancestors to critically evaluate the burial environment and taphonomic history of a burial during the period of time it transitioned from the biosphere to the geosphere before excavation by archaeologists.

## Author Contributions

**Melanie M. Beasley:** conceptualization (lead), formal analysis (lead), writing – original draft (lead), writing – review and editing (lead).

## Supporting information


**Data S1.** Supporting Information.

## Data Availability

The raw data for the study has been previously published (Bartelink et al. 2020; Beasley et al. 2014, 2024; Wieberg 2010) and compiled in Supporting Information Dataset.
